# Surgical Management of a Large Congenital Melanocytic Nevus of the Face—A Technical Case Report and Comparison with Classic and Novel Approaches

**DOI:** 10.3390/pediatric18040085

**Published:** 2026-06-25

**Authors:** Kostadin Gigov, Petra Kavradzhieva, Ivan Ginev, Mihaela Prandzheva, Mariya Miteva

**Affiliations:** 1 Section of Plastic Reconstructive and Aesthetic Surgery and Thermal Trauma, Department of Propedeutics of Surgical Diseases, Faculty of Medicine, “St. George” University Hospital Plovdiv, Medical University of Plovdiv, “Peshtersko Shausse Blvd 66”, 4002 Plovdiv, Bulgaria; kostadin.gigov@phd.mu-plovdiv.bg (K.G.); ivan.ginev@mu-plovdiv.bg (I.G.); 2 Medical Faculty, Medical University of Plovidv, Vassil Aprilov Blvd 15A, 4002 Plovdiv, Bulgaria; 21101058@mu-plovdiv.bg; 3 Department of Endocrinology and Metabolic Diseases, Faculty of Medicine, “St. George” University Hospital Plovdiv, Medical University of Plovdiv, Vassil Aprilov Blvd 15A, 4002 Plovdiv, Bulgaria; m_miteva@yahoo.com

**Keywords:** large congenital melanocytic nevus, staged excisions, tissue expansion, pediatric reconstructive surgery

## Abstract

Large congenital melanocytic nevi (LCMN) of the face can pose significant functional, esthetic, and psychosocial challenges in childhood. In selected patients, staged excision offers a practical reconstructive strategy when primary closure is not feasible without distortion of nearby facial landmarks. We report the management of a child with a facial LCMN using a planned multistage surgical approach aimed at lesion removal while preserving contour and minimizing scar burden. The lesion was excised sequentially over three procedures, with careful attention given to relaxed skin tension lines and facial esthetic units. When required, adjunctive reconstruction was performed to optimize closure and support tissue healing. This approach allowed a gradual reduction in the nevus, improved tissue accommodation, and avoidance of excessive tension on the surrounding skin. Postoperative recovery was uncomplicated, and the final esthetic outcome was satisfactory for both the patient and parents. Staged excision was selected over tissue expansion and skin grafting because it allowed progressive lesion reduction while preserving adjacent facial landmarks and minimizing donor-site morbidity. This technical case highlights the importance of individualized surgical planning, preservation of facial esthetic units, and staged scar placement when managing large facial congenital melanocytic nevi in pediatric patients. The educational value of the report lies in illustrating the decision-making process used to balance lesion removal, esthetic outcomes, and long-term surveillance in a challenging facial location.

## 1. Introduction

Large congenital melanocytic nevi (LCMN) are benign pigmented lesions present at birth, arising from abnormal proliferation of melanocytes during embryonic development, which can affect not only the skin, but also present with extracutaneous abnormalities. Large lesions have an increased but variable risk of developing melanoma, and can show diverse clinical presentations [1,2]. A common genetic change in LCMN affects the NRAS gene and is thought to induce excessive proliferation of melanocytic lineage cells, leading to nevus development. Dysfunction of the cytokine hepatocyte growth factor, which controls the migration and growth of melanocytes, has also been associated with this disorder. Although there is some evidence suggesting a familial contribution, most LCMNs are thought to arise from sporadic mutations [3,4,5].

LCMN lesions located on the face may create significant cosmetic and psychosocial concerns, particularly in children, because they are highly visible and may affect self-image, social interaction, and parental anxiety. Management of facial LCMN is individualized and depends on the size, anatomical location, expected growth, potential for functional impairment, and family preference. Surgical excision remains a mainstay of treatment, and in larger or more complex lesions, staged excision is often preferred to achieve better esthetic outcomes while preserving adjacent structures and minimizing tension on wound closure and avoiding the burden of a prolonged hospital stay [4,5,6,7].

We describe a technical case report of a 4-year-old girl with a congenital melanocytic lesion involving the entire left preauricular and temporal region, as well as the entire left superciliary area. Because of the extent and distribution of the nevus, complete removal in a single operation was not feasible without risking distortion of the surrounding facial units. For this reason, a staged surgical approach was chosen, and the lesion was managed over three excisions. This strategy allowed progressive reduction in the nevus burden while maintaining acceptable cosmetic and functional results.

After the last excision, the only remaining portion of the lesion was confined to the superciliary region. At present, the parents have elected to defer further surgical intervention, and no additional procedures are planned in the immediate period. Future management of the brow lesion will be reconsidered later, with possible use of modern reconstructive methods depending on the child’s age, tissue characteristics, and family wishes. Although staged excision is a well-established reconstructive technique, reports describing its application in extensive pediatric facial LCMN, involving multiple esthetic units with long-term staged planning, remain limited. The educational value of the present technical case lies in demonstrating the surgical decision-making process, scar-planning strategy, preservation of facial landmarks, and family-centered management during long-term treatment [7].

## 2. Materials and Methods—Technical Case Presentation

This technical case report describes the surgical management of a 4-year-old girl with a large congenital melanocytic nevus, involving the entire preauricular region, the left temporal region, and the left superciliary area. After clinical assessment and preoperative planning, a staged excision approach was selected because the extent and location of the lesion made complete removal in a single operation impractical without risking excessive wound tension, distortion of adjacent facial structures, and an unfavorable cosmetic result. Staged excision is an established reconstructive strategy for selected facial LCMNs, particularly when preservation of facial contour and esthetic units is a priority.

**Figure 1:**
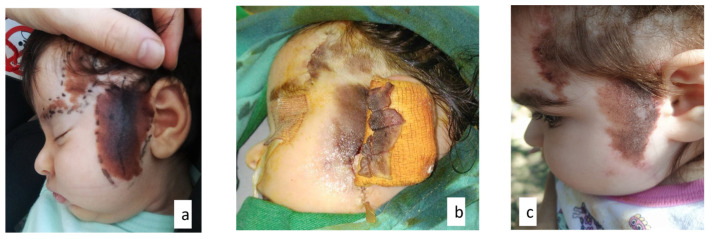
(**a**) Preoperative markings for the first intervention of the planned staged-excision approach. (**b**) Intraoperatively showcasing the excision of more than 1/3 of the nevus. (**c**) Six months after the first intervention.

**Figure 2:**
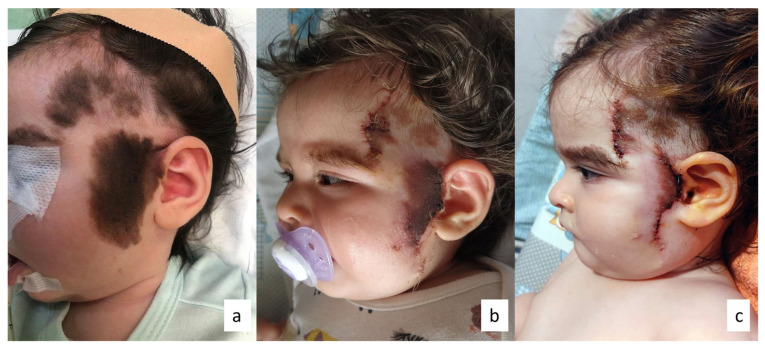
(**a**) Prior to first surgery; (**b**) first postoperative day after first surgery; (**c**) first postoperative day after second excision. The interval between the first and second surgeries was 9 months.

**Figure 3:**
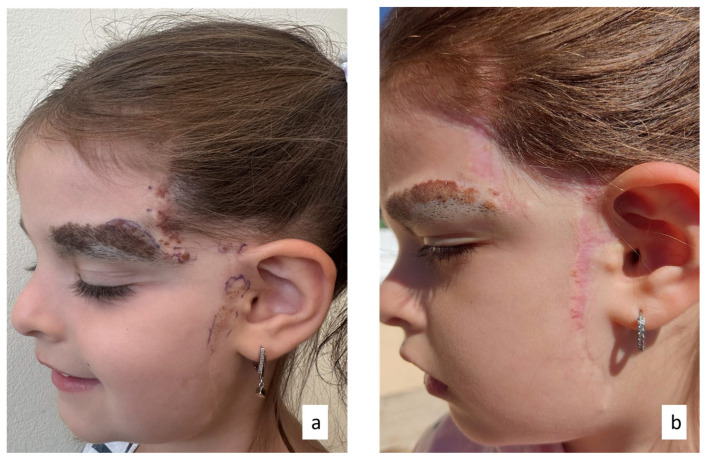
(**a**) Excision markings prior to the third operation; (**b**) one year after the third surgery.

The lesion was managed over three operative stages under general anesthesia. At each stage, a portion of the nevus was excised, and incision lines were strategically placed to preserve the esthetic subunits of the face. This approach allowed adequate tissue relaxation and maintained sufficient local anatomy for subsequent tension-free closure. Surgical planning focused on the preauricular, temporal, and brow regions, with careful attention given to the natural boundaries and minimization of visible scarring. Curvilinear, crescent-like incisions were made along vectors that avoid distortion and traction of the lateral canthus, eyebrow, auricle, and hairline. This is a major consideration in pediatric plastic surgery, as even moderate tension can cause significant distortion and further asymmetry with growth. Incision lines followed relaxed skin tension lines in the preauricular region and in the temporal area, aimed at preserving the hairline intact. The undermining plane was developed in the Superficial musculoaponeurotic system (SMAS) layer. At the first stage, more than one-third of the nevus was excised (Figure 1), or approximately 40%, with a vast dissection extending approximately 1 cm anteriorly to the lesion in the preauricular area; at the second stage(Figure 2b,c), nearly 30%, and at the third stage, around 10% was excised (Figure 3). Subcutaneous and intradermal sutures were placed. Between procedures, healing and tissue adaptation were allowed to optimize conditions for the next excisions. The first stage was performed when the child was 11 months old. The interval between the first and second stage was nine months, and between the second and the third was one year.

**Figure 4:**
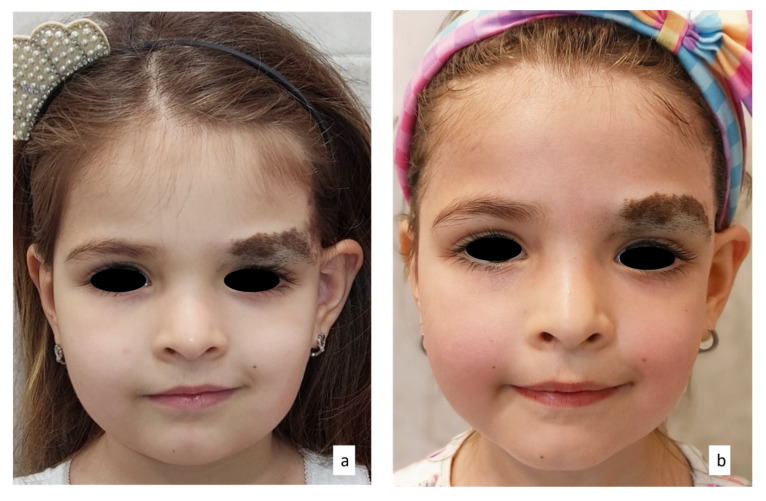
(**a**) Prior to the third intervention; (**b**) one year after the third intervention. Scars are strategically placed to resemble the healthy side.

Following the third excision, the only residual component of the lesion remained in the superciliary region (Figure 4). At that point, the parents elected to defer further surgical treatment, and no additional intervention was undertaken. Future management of the remaining brow lesion was postponed for later consideration, including the possible use of modern reconstructive or minimally invasive techniques, such as ablative lasers, depending on the child’s age, tissue characteristics, and family preference. This approach reflects the individualized nature of LCMN and the balance between surgical radicality and family-centered decision-making. No postoperative complications were observed at any stage, and each procedure required a 2-day hospital stay. After each excision, histological examination was performed. Histopathological findings remained consistent across all stages, identifying an intradermal nevus without signs of atypia, with Ki-67 expression confirmed to be less than 3% after each excision. After the final excision, the margins were confirmed to be clear for the preauricular and temporal areas. No deep and junctional components were identified. Future management of the residual nevus includes regular follow-ups every 3–6 months, with photodocumentation and dermoscopy. Planned treatment comprises excision of the superciliary component with histopathological examination, followed by skin grafting, tattooing, or micropigmentation. Laser treatment remains a possible adjunctive option.

**Figure 5:**
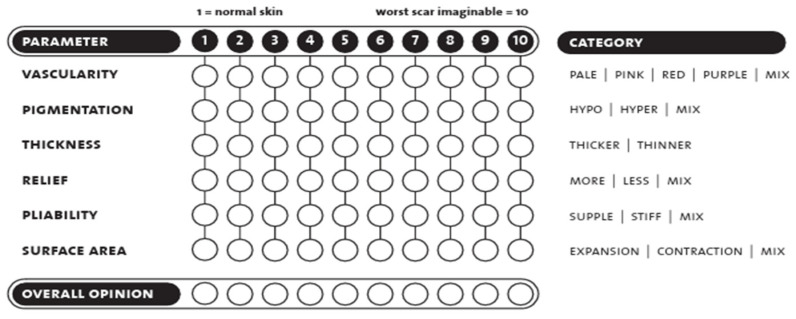
POSAS 2.0 scale.

Scar quality was evaluated by using a validated tool for observation, Patient and Observer Scar Assessment Scale (POSAS) 2.0 [8] (Figure 5). The POSAS 2.0 Observer Scale was completed by the treating surgeon. Individual domain scores were as follows: vascularity 1, pigmentation 2, thickness 1, relief 1, pliability 1, and surface area 2, resulting in a total Observer Scale score of 8 (with a minimum possible score = 6). Patient- or parent-reported POSAS assessment was not collected and is acknowledged as a limitation of this report.

## 3. Discussion

Contemporary management of large congenital melanocytic nevi (LCMN) in children is increasingly individualized and multidisciplinary, with staged excisions and tissue expansion remaining the main reconstructive approaches, and lasers used selectively for debulking, blending residual pigmentation, and improving cosmesis rather than for melanoma-risk reduction [6,7,8,9].

The modern approach begins with careful characterization of the lesion size, location, color heterogeneity, surface features, number of satellite lesions, and the possible presence of neurocutaneous melanocytosis. Large lesions often require complex multidisciplinary assessment, involving pediatric dermatology, plastic surgery, radiology, neurology, orthopedics, and psychological support, especially when the lesion is extensive or located on cosmetically and functionally sensitive areas [10].

Observation remains acceptable in selected children, particularly when surgery is highly morbid or unlikely to remove most of the nevus burden. However, many giant lesions are treated actively in a surgical manner because of the psychosocial impact, caregiver concerns, difficulty with surveillance, and the practical challenge of managing very large, pigmented areas over time [10].

Among operative options, staged or serial excision and tissue expansion remain the principal reconstructive approaches for many giant lesions. Both aim to remove as much nevus tissue as feasible while replacing the defect with adjacent skin of similar quality; however, they differ in execution, morbidity profile, and indications [11,12].

### 3.1. Staged Excision

Staged excision, also called serial excision, involves planned partial resections with direct closure at each stage. The method relies on the inherent elasticity and creep of the surrounding skin, which is often favorable in children. Procedures are spaced over months, allowing tissue to relax before the next resection. This technique is especially useful on the trunk and limbs, where skin laxity may permit a substantial reduction in lesion size over two to several operations without the use of tissue expanders or grafts [10].

The advantages of staged excision include avoidance of a foreign body, shorter individual operations, no need for repeated expander inflation visits, a relatively straightforward postoperative course, and shorter hospital stays. Postoperative care includes frequent dressing changes, which is a relatively lower burden of care compared to tissue expanders. It also allows progressive histopathologic examination of excised tissue and can produce acceptable linear scars when carefully planned along tension lines. For lesions that can be removed in a limited number of stages, serial excision is often considered efficient and predictable [7].

Its limitations are equally important. Very large lesions may require many operations, and closure becomes progressively more difficult in anatomic regions with less skin laxity. Scar widening, contour distortion, dog-ear formation, and islands of residual nevus may remain, especially if the nevus extends widely beyond the zone that can be mobilized safely. On the scalp or face, staged excision may be less attractive if it sacrifices hair-bearing skin or distorts important esthetic units [7,10].

### 3.2. Tissue Expansion

Tissue expansion approaches the problem in a completely different manner compared to staged excision. It is a well-established surgical approach to grow skin continuously in the course of a couple of months, applied in the management, not only for the removal of large and giant melanocytic lesions, but also for conjoined twins, as well [11]. A tissue expander is placed in the subcutaneous plane and gradually inflated over time, creating additional local tissue that can later be advanced or transposed after nevus excision. This technique is particularly valuable in the scalp, face, and neck, where tissue match is crucial and where preserving hair-bearing skin or facial subunits has major cosmetic value [12].

The main strength of tissue expansion is that it generates replacement skin with similar color, thickness, adnexal structures, and texture. In scalp reconstruction, this can preserve hair density and direction better than grafting or more aggressive serial excision. For very large lesions, expansion may also reduce the total number of definitive excisional stages by providing more skin in a controlled way [13,14].

However, tissue expansion is associated with a distinct burden of care. It requires implantation of a foreign device, repeated clinic visits for inflation, and a temporary period of visible skull contour deformity that some children and families find distressing. Complications include infection, seroma, hematoma, expander rupture, wound dehiscence, ischemia, flap necrosis, and expander extrusion or device port complications, any of which may lead to premature removal and could compromise the reconstructive plan [10,13]. Another important aspect in pediatric tissue expansion is excessive thinning of the scalp compared to an expansion in adults [11]. In addition, because of potential effects on skull development, including fontanelles and suture widening, tissue expansion should be carefully considered, and in most cases, avoided until 12–18 months of age [11].

For lateral face reconstruction, expansion of the neck is a practical and applicable approach to create a Mustarde cheek rotation flap to cover the defects [11,15]. However, this might result in a more visible scarring with significant widening with facial growth and change, compared to the staged excision we used in our patient.

When staged excision and tissue expansion are compared, neither method is predominantly superior. Serial excision tends to be favored when the nevus is located on areas with good laxity and can be removed in a few stages without severe distortion. Tissue expansion is often favored when a lesion is too large for direct closure or when esthetic unit preservation is central, especially on the scalp and face [13,14].

### 3.3. Skin Grafting

Skin grafting is another classic approach used in the treatment of large congenital melanocytic nevi [16]. It offers a significant advantage over other techniques because it can often be performed in a single stage. However, major disadvantages and complications are associated with this method, such as donor-site morbidity, significant graft contracture, hematoma, infection, and marked mismatch between the texture and color of the graft and the surrounding skin. Also, in pediatric facial reconstructive surgery, this is usually a secondary option for treatment [17].

### 3.4. Laser Treatment

Laser treatment currently occupies a more limited but important and emerging position in the contemporary management of giant congenital melanocytic lesions. Current reviews generally describe laser therapy as an adjunctive or cosmetic modality rather than a definitive oncologic treatment. This position reflects the biological reality that nevus cells often extend deeply into the dermis, around adnexal structures, and into subcutaneous tissues that are not fully eradicated by most laser systems [10].

Several laser types have been used in congenital melanocytic nevi, including Q-switched ruby, alexandrite, and Nd:YAG lasers for pigment targeting, as well as ablative carbon dioxide and Er:YAG lasers for resurfacing or debulking. These modalities can reduce visible pigmentation, improve texture, and flatten selected raised components, but they do not reliably remove all nevus cells, and recurrence or repigmentation is well recognized [10,18].

Laser treatment of residual nevus after surgery is one of the more defensible indications. Following serial excision or expansion-based reconstruction, small remaining pigmented islands, patchy discoloration, or textural irregularities may persist at the margins or within scars. In that setting, laser therapy may improve the cosmetic result without attempting to replace surgical excision as the primary treatment [18].

Pigment-specific lasers are mainly used for residual superficial pigmentation. Their effect is usually better when the pigmented component is relatively superficial and less effective when deeper dermal nevus cells dominate the lesion. Ablative resurfacing lasers such as carbon dioxide or Er:YAG can also be used to smooth elevated areas or blend scar-nevus interfaces, although this must be balanced against the risk of scarring and dyspigmentation [18].

Reports of Er:YAG laser resurfacing in neonates and infants with large congenital nevi suggest that substantial lightening and acceptable cosmetic results may be achieved in selected cases. More recent reviews continue to stress that laser-induced improvement in appearance should not be equated with complete biological eradication of the lesion [18].

One of the main controversies surrounding laser treatment is surveillance. Partial destruction of a congenital nevus can create fibrosis, irregular pigment patterns, and altered clinical landmarks that may make later examination more difficult compared to staged excision. Because of this concern, some authors caution against the routine use of lasers as primary therapy for large lesions when the main objective is oncologic reassurance [18]. Adverse effects of laser therapy in children include transient erythema, edema, crusting, post-inflammatory hyperpigmentation or hypopigmentation, and, less commonly, scarring or infection. The risk profile varies by wavelength, fluence, body site, skin phototype, and whether the treatment is ablative or nonablative [18].

The aforementioned approaches have been described for large facial congenital melanocytic nevi, each with distinct advantages and limitations. A comparison of the most commonly employed approaches is presented in Table 1.

### 3.5. MAPK Pathway Inhibitors as a Future Therapeutic Approach

Recent studies have moved toward precision therapies aimed at molecular drivers, such as inhibitors of the Mitogen-activated protein kinase (MAPK) pathways, RNA-based therapies that reduce MAPK signaling and induce apoptosis in LCMN cells in vitro, and topical immunotherapy with Squaric acid dibutyl ester (SADBE) hapten, showing promising results in mouse models, acting through macrophage activation [19]. Although these approaches are novel, the data are still in the preclinical phases. LCMNs carry a low but definite risk of melanoma, which is highest for the largest lesions, and of particular concern when the face is affected, as it is the area that experiences the greatest sun exposure [19,20]. Though emerging targeted therapies show promising regression of NRAS/BRAF-driven LCMN in preclinical models and isolated clinical reports, they remain under investigation and are still questionable as to whether they completely eliminate deeper and extracutaneous melanocytic cells in vivo [20,21]. On the contrary, surgical excision currently offers the most definitive (and within a relatively shorter time frame) treatment, with the most definitive debulking of melanocytic tissue in operable giant lesions.

Staged excision was our first choice of treatment as it provides controlled and consistent observation of the histopathological profile of the nevus. The burden of care is lower, and the hospital span is shorter. This is a significant advantage, which takes into account the psychological perspective. In comparison to tissue expansion, staged excision does not involve foreign body reactions as a complication, the bleeding control is more precise, and there is no contracture of the skin compared to skin grafting. Staged excision can be combined with future laser treatment, additional excision within the superciliary region, followed by tattooing methods or future local immunotherapy.

## 4. Conclusions

Overall, the contemporary treatment of large congenital melanocytic lesions in children is individualized and staged over time. The approach should be multidisciplinary, and various modalities can be incorporated as a whole therapeutic plan. Staged excision remains attractive for lesions that can be progressively removed with direct closure and acceptable scarring, while tissue expansion is particularly valuable when a large amount of matched skin is required, especially in the scalp and face. In our patient, staged excision resulted in an esthetic appearance, with scars strategically placed without any disfigurement, a reduced burden of care, and a positive psychosocial impact. However, active surveillance of the residual part of the nevus is obligatory, as melanoma risk is not eliminated. Lastly, newer, less invasive approaches like laser procedures or topical immunotherapy show promising results.

## Figures and Tables

**Table 1:** Comparative chart of the treatment approaches.

Treatment Approach	Principle	Advantages	Disadvantages	Esthetic Outcome	Complications
Serial (staged) excision	Multiple excisions of portions of the nevus, relying on skin creep and laxity for primary closure.	-Simple technique -No foreign body insertion and reaction -No donor-site morbidity -Can be performed as an outpatient surgery. -Continuous histopathological examination	-Multiple surgeries -Prolonged treatment -Limited by lesion size and skin laxity	-Good to excellent outcome when aligned with relaxed skin tension lines and facial subunits	-Scar widening and hypertrophy -Wound dehiscence -Distortion of facial units -Hematoma
Tissue expansion	Placement of a subcutaneous expander adjacent to the nevus, followed by a gradual inflation of the device to generate excessive skin	-Adjacent skin color, texture, and thickness matching -Possible complete excision at one stage with histopathological examination	-Multiple procedures for inflation -Foreign body reaction -Prolonged hospital stays -At least two surgeries	-Good to excellent outcome when scars are aligned with skin tension lines -Excellent match with adjacent skin color and texture	-Expander exposure or extrusion -Infection -Device port complication -Flap ischemia -Psychological distress due to highly visible disfigurement -Skull deformities -Hematoma
Skin grafting	Complete or partial excision of the nevus followed by coverage with split- or full-thickness skin grafts	-Allows immediate removal of a large lesion in a single stage with reconstruction -No expansion periods -Usually a single surgery	-Poorest color, texture, and thickness match -Graft contracture -Donor-site morbidity	Usually inferior to tissue expansion and staged excision, especially on the face	-Partial or complete graft loss -Hematoma -Distortion in highly functional zones (e.g., ectropion) -Hypertrophic scarring -Pigment mismatch
Lasers	Selective destruction of nevus cells using lasers, such as Q-switched ruby, alexandrite, Nd: YAG, or ablative Co2 lasers	-Minimally invasive -Avoids large scarring -Can improve cosmetic outcomes	-No reliable removal of melanocytic tissue -No histopathological verification -Higher recurrence rate -Multiple sessions -Persistent malignant risk	Variable	-Hypo- or hyperpigmentation -Incomplete removal -Difficulty in future surveillance

## Data Availability

Data sharing is not applicable to this article.

## Abbreviations

The following abbreviations are used in this manuscript:

LCMN	Large congenital melanocytic nevus
SMAS	Superficial musculoaponeurotic system
MAPK	Mitogen-activated protein kinase
POSAS	Patient and Observer Scar Assessment Scale
SADBE	Squaric acid dibutyl ester
